# A multicriteria resource allocation model for the redesign of services following birth

**DOI:** 10.1186/s12913-018-3430-1

**Published:** 2018-08-22

**Authors:** John Bowers, Helen Cheyne, Gillian Mould, Martin Miller, Miranda Page, Fiona Harris, Debra Bick

**Affiliations:** 1 Stirling Management School, University of Stirling, Stirling, FK9 4LA UK; 2 Nursing, Midwifery and Allied Health Professions Research Unit, Unit 13 Scion House, Stirling University Innovation Park, Stirling, FK9 4NF UK; 3 Florence Nightingale School of Nursing and Midwifery, King’s College London, James Clerk Maxwell Building, 57 Waterloo Road, London, SE1 8WA UK

**Keywords:** Postnatal care, Maternity services, Cost savings, Care quality, Resource allocation, Priority setting, Program budgeting and marginal analysis, Multicriteria decision analysis

## Abstract

**Background:**

Many healthcare services are under considerable pressure to reduce costs while improving quality. This is particularly true in the United Kingdom’s National Health Service where postnatal care is sometimes viewed as having a low priority. There is much debate about the service’s redesign and the reallocation of resources, both along care pathways and between groups of mothers and babies with different needs. The aim of this study was to develop a decision support tool that would encourage a systemic approach to service redesign and that could assess the various quality and financial implications of service change options making the consequent trade-offs explicit. The paper describes the development process and an initial implementation as a preliminary exploration of the possible merits of this approach.

**Methods:**

Other studies have suggested that combining multicriteria decision analysis with programme budgeting and marginal analysis might offer a suitable basis for resource allocation decisions in healthcare systems. The Postnatal care Resource Allocation Model incorporated this approach in a decision support tool to analyse the consequences of varying design parameters, notably staff contacts and time, on the various quality domains and costs. The initial phase of the study focussed on mapping postnatal care, involving interviews and workshops with a variety of stakeholders. This was supplemented with a literature review and the resultant knowledge base was encoded in the decision support tool. The model was then tested with various stakeholders before being used in an NHS Trust in England.

**Results:**

The model provides practical support, helping staff explore options and articulate their proposals for the redesign of postnatal care. The integration of cost and quality domains facilitates trade-offs, allowing staff to explore the benefits of reallocating resources between hospital and community-based care, and different patient-categories.

**Conclusions:**

The main benefits of the model include its structure for assembling the key data, sharing evidence amongst multi-professional teams and encouraging constructive, systemic debate. Although the model was developed in the context of the routine maternity services for mothers and babies in the days following birth it could be adapted for use in other health care services.

**Electronic supplementary material:**

The online version of this article (10.1186/s12913-018-3430-1) contains supplementary material, which is available to authorized users.

## Background

### Postnatal care in the NHS and the need for an option appraisal tool

The National Health Service (NHS) in the United Kingdom (UK) faces continuing growth in demand while budgets remain tightly constrained. Many services are being redesigned with no substantial analysis of the likely effects on the quality of care or the demand for NHS resources. Typically service redesign in the NHS is reactive, based on implicit heuristic assumptions about potential savings and care quality; there is a need for a more systematic approach. Redesign often focusses on reallocating resources with a particular emphasis on moving care from acute hospital settings to the community [1]. The rationale is that reducing the length of time spent in hospital releases resources and reduces costs [2], while quality is increased by diverting resources to provide more care in the community [3]. In this paper we present a service redesign tool that attempts to provide a comprehensive appreciation of the quality and financial consequences of redesign options. The tool was developed in the context of the routine UK NHS maternity services for mothers and babies in the days following birth but it might be adapted for use in other health care services. Indeed the design of the tool was based on earlier work examining the balance of acute and community care in a variety of services [4].

Postnatal care in the UK is a high volume, universal service provided to all mothers and their babies for a minimum of 10 days after the birth, first in hospital and then in the community. In recent years postnatal in-patient stays have declined substantially, mainly motivated by a desire to reduce costs but also in response to many women’s desire to return home more quickly. In 1990, most women remained in hospital for at least three days after giving birth [3]; by 2013 over 80% were discharged home within 48 h [2, 5]. This reduction in hospital-stay has not been accompanied by additional investment in the community services. While mothers in the UK used to receive daily midwife visits at home for 10 days after the birth [4], by 2013 78% had just 1–5 postnatal staff-contacts [5]. Successive surveys of UK women’s experiences have indicated that satisfaction with their postnatal care is poor compared with other aspects of maternity care (see Additional file 1 NHS Scotland Maternity Care Survey). A substantial minority of mothers report receiving inconsistent advice and insufficient support on issues such as infant feeding and their own physical and emotional recovery [6, 7]. Both the traditional UK pattern of daily midwife home visits and the more recent delivery-model of short inpatient stay with reduced community midwife contacts often adopt a ‘*one-size fits all’* approach based on organisational priorities, rather than recognising the needs of individual women [8]. This can result in a poor allocation of resources with some mothers and babies receiving more visits than necessary while others may not have their care needs met [9].

Other studies have examined individual service changes in postnatal care and their consequences in isolation. The aim of this study was to develop a decision support tool, the Postnatal care Resource Allocation Model (PRAM), that would encourage a more systemic approach. The objective was to produce a tool that could assess the various quality and financial implications of options consisting of packages of service changes, and make the consequent trade-offs explicit. The model exploited a variety of evidence from academic literature, case studies, service level audits such as the NHS Scotland workforce planning study [10] as well as stakeholders’ experiences captured in a series of workshops and interviews. In this paper we describe and discuss the PRAM development process and its initial implementation in one NHS site.

### Option appraisal and resource allocation models

Many of the more contentious issues in the redesign of any healthcare service relate to the allocation of resources, both between different categories of patients and along the care pathways. Priority setting approaches such as programme budgeting and marginal analysis (PBMA) have been employed successfully in many redesign applications [11–13] but they often require substantial input from staff acting as panel members and this can hinder implementation [14, 15]. A full PBMA entails much debate and data collection, re-examining all assumptions and data. This can be useful but it does require considerable time and commitment from clinical and managerial staff who have other competing priorities. Timeliness is often vital in ensuring successful implementation of priority setting and resource allocation tools and in some redesign exercises it may be desirable to short-circuit stages of the PBMA process. A particular difficulty arises with some quality dimensions which cannot be readily quantified. The analysis must then employ more qualitative evidence, or exclude key criteria: “economic evaluation often fails to present dimensions that appear important for decision-makers, such as equity” [16]. In many applications a focus on cost effectiveness is not sufficient; a wider range of criteria are needed to reflect stakeholders’ concerns, though there may be much debate about their relative importance [17–19]. In response to this need for a flexible approach accommodating diverse forms of evidence, some studies have adopted multicriteria decision analysis (MCDA) incorporating diverse information exploiting both rigorous quantitative data and qualitative expert judgement [20–23]. This flexibility can encourage wider engagement and a better appreciation of diverse stakeholders’ views as part of a more deliberative approach [19]. It has been suggested that a combination of PBMA and MCDA might provide a practical, flexible basis for resource allocation [12, 24]; this combination provided the basis for the design of the PRAM decision support tool developed in this study.

The core of PRAM is a model of the relationships between the design parameters, cost and quality. Developing this model completely afresh in each application, as in many examples of PBMA and MCDA, can be a most valuable exercise and produce a deeper appreciation amongst the stakeholders. However, a major objective of the current project was to develop a tool that could be deployed relatively easily in a timely manner. Hence this study developed a generic set of relationships relevant in a wide range of applications in postnatal care, though with the scope for adaptation to accommodate the local context. As part of this more generic approach, the tool adopted the quality domains specified by the Institute of Medicine [25], interpreted in the context of postnatal care.

## Methods

### Developing the model

The initial phase of the development focussed on mapping current postnatal care, involving case studies in three NHS organisations. The case study sites were chosen to reflect geographical, socio-economic and ethnic diversity. Group discussions, and some individual interviews, were undertaken in each site. These involved representatives of all postnatal care and service management staff working in both the hospital and community including: midwives, maternity service leads, heads of midwifery, maternity care assistants, obstetricians and members of maternity care liaison committees representing service users. Discussion focussed on eliciting current postnatal care pathways and the barriers and enablers to provision of high quality postnatal care (see Additional file 2 Developing pathways). This was an iterative process with between four and seven group meetings at each site. Initial discussions and interviews allowed pathway maps to be drafted; these were then reviewed and refined in subsequent meetings. Following the principles of Experienced Based Design [26], additional survey data were collected and integrated into the pathways (see Additional file 3 EBD questionnaire). Postal questionnaires were used to assess women’s experience and wellbeing, adapting the Care Quality Commission maternity survey [27] with additional questions on maternal postnatal health and the Edinburgh Postnatal Depression Scale (see Additional file 4 Postnatal care survey). These were distributed to women six weeks following birth, with 351 women responding (response rate 31%). A small subgroup of 9 women also participated in individual interviews in which they were asked to describe their experience of postnatal care in relation to key points on the postnatal care pathway. These data were supplemented with a set of literature searches to collect evidence of the effects of alternative approaches to postnatal care. The literature searches were structured around key topics identified from the NICE [8] and other robust clinical guidance, notably relating to ‘where, who, when’ aspects of care for example, timing of hospital discharge, location of care, roles of maternity care assistants, continuity of carer. The quality of the resultant evidence was ranked using criteria from NICE guidance and summarised as a preliminary knowledge base.

**Fig. 1:**
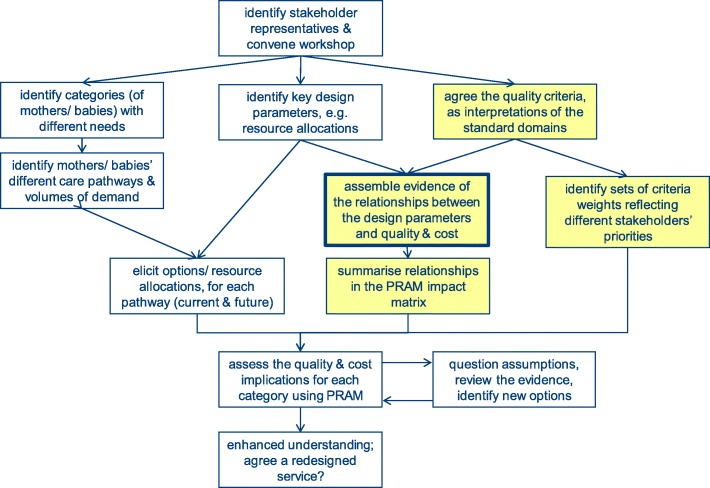
The PRAM process: assembling data and assessing options

A wide variety of evidence, from case studies and literature was assembled describing the relationships between key design parameters and their impact on quality and cost. The evidence was shared and tested through a series of four workshops with participants including service users, health policy makers, midwives, service managers, perinatal mental health staff and general practitioners (see Additional file 5 Exploring assumptions). Typically each workshop stimulated further research to identify additional evidence to confirm or adapt the model’s knowledge base. This assembly of evidence formed the core of whole PRAM process, as highlighted in Fig. 1. The decision support tool was based on the principles of PBMA and MCDA and encoded in Excel to help ensure its accessibility. Prototype screens were shared with the potential users and the model refined to ensure that the tool clearly addressed their concerns and the possible redesign options (see Additional file 6 Prototype testing).

The model included three key features:

- High level pathways for each of four categories of mothers and babies. The tool was intended to reflect the different needs in these categories and to achieve a greater degree of equity, while striving for overall cost-effectiveness [28]. The pathways provide a structure for the data input, notably the number and nature of the staff contacts, and the resource requirements of each activity during both hospital and community-based care. These are illustrated in Tables 3 and 4 which consider two options: A, the current provision, and B, a proposed redesigned service.
- The impact matrix summarising the relationships between the design parameters, notably the resource allocations, and the quality domains. Financial models were also incorporated to provide a comprehensive assessment of quality and cost. The impact matrix considers the effects of separate design parameters but is otherwise similar to the performance matrix employed in some MCDA applications [23].
- A set of outputs indicating the relative effects on the decision criteria, notably the Institute of Medicine quality domains [25] and costs, for each of the four categories of mother and baby. A summary aggregates the separate quality domains’ scores using weights to reflect their relative priority.

Figure 1 illustrates the complete PRAM implementation process. Some activities, shaded in Fig. 1, are preparatory and are based on the generic data, with limited local staff input to confirm or adapt the assumptions to ensure their relevance. The other activities involve more substantial local input, both data describing volumes of activity and also workshops with selected stakeholders to understand the local context, concerns and redesign options. Typically this is an iterative process, considering the outputs and revising the options before arriving at an agreed redesign for the service, as illustrated in Fig. 1. The number of iterations may vary, dependent on the time available and the magnitude of the redesign, but typically there are three phases. In the first phase, a PRAM workshop may be regarded as a training exercise and a review of the data, prompting discussion about key inputs and identifying any further data requirements. The second phase focusses on exploring the major redesign options. This can involve several workshops and iterations as the stakeholders develop a fuller appreciation of the real problems and a shared understanding of the complete postnatal care system. In the final phase, further iterations may be needed to refine a particular option until a chosen redesign specification is agreed. This final phase may involve just a few key staff rather than full workshops. The iterative process can encourage a greater engagement of a wider range of staff leading to an “evidence informed deliberative process” [19, 26, 29–31]. Such an approach helps increase the perceived legitimacy of the resultant redesign, enhancing the chances of successful implementation [16].

### Identifying categories of need and the care pathways

At the point of care it is important to identify individual mother and baby’s needs. However, the objective of the PRAM project was to assess redesign options at the overall service planning level. Table 1 summarises the broad categories of need used in this study, corresponding to the acuity levels employed in NHS studies [10]. Initial trials experimented with many more categories and more detailed, condition-specific descriptors. However, this became unwieldy and it was agreed that a smaller set of categories using high level descriptors was more practical. Where necessary these descriptors can be adapted to reflect the local context and terminology. Other PRAM applications might consider the value of further categories reflecting mothers’ different needs; mothers of different cultural backgrounds may have particular requirements that imply extra support during some stages of postnatal care.

**Table 1:** Defining the care categories

Category	Definition
0a	Routine care plus parenting support for healthy mother and baby but lacking parenting/ feeding skill or confidence.
0b	Routine care plus parenting support for healthy mother and baby but lacking parenting/ feeding skill or confidence.
1	Additional care for mother and /or baby with some medical, mental health and /or social needs.
2	Additional care with liaison with other services to meet complex health or social needs.
3	Intensive additional care with liaison with other services to address serious, complex health or social needs. Given the very low numbers in this category they were subsumed into a joint category 2&3.

Nearly all mothers in the UK receive maternity care in hospital after giving birth and then in the community following national guidance [8]. However, the staff-contacts and resource inputs should reflect the needs of different categories of mothers and babies. Tables 3 and 4 provide an example of the data specifying the allocation of resources for each of the four categories of mothers and babies. The key design parameters span:

- Hospital-based care, as illustrated in Table 3, including length of stay; number of staff; staff mix; feeding and parenting support.
- Community-based care, as in Table 4, including number, nature and duration of contacts with NHS staff; staff mix; feeding and parenting support.

### Defining quality criteria in relation to postnatal care

One of the key steps in MCDA is the selection of decision criteria [12, 23, 30]. There was considerable debate about these criteria, with some advocating the use of very localised criteria. Eventually, it was agreed that the PRAM criteria should be based on the standard international quality domains [25] since this increases the scope for more general understanding and application in diverse locations and care environments. However, it was necessary to interpret the standard domains in the specific context of postnatal care, as in Table 2. Various approaches to incorporating costs have been adopted in option appraisal and resource allocation but all can be problematic [18, 23]. PRAM separates cost and quality presenting the assessment in a form that helps decision makers debate quality-cost trade-offs while avoiding the controversies of an explicit cost-benefit analysis with its requirement for monetary values to be attributed to each quality domain.

**Table 2:** Interpreting the quality domains in postnatal care

Domain	Interpretation in postnatal care	Weight
Safe	Avoidance of care associated harms; care not delivered as planned, inconsistent or variable care	0.50
Effective	Supporting recovery from birth and physical health & mental well being for mothers & babies	0.30
	supporting development of confidence in parenting	
Timely	Information provided at the appropriate stage to support parents, e.g. infant-feeding to encourage good practice from an early stage	0.05
Equitable	Equal access to care, e.g. regardless of physical ability or geography	0.05
Person-centred	Individual care plans reflecting the mother & baby’s needs, e.g. home/clinic visits designed around mothers’ preferences	0.10

Although it can be valuable to examine each quality domain separately, an aggregated measure is also useful when comparing options and developing an appreciation of the quality-cost trade-offs [32]. This aggregation requires explicit weights for each of the quality domains, as included in Table 2. Since the decision structure employed just five quality criteria, a simple approach was adopted to specifying the weights based on discussions in the workshops with a range of staff including midwives, service managers, GP’s and service users. The stakeholders were encouraged to propose a variety of sets of weights representing diverse views, as part of a more inclusive, deliberative approach [19]. Some stakeholders initially argued that only safety and effectiveness should be weighted. However, eventually it was generally accepted that each criterion could be important and deserved some representation with a non-zero weight and a reasonable consensus was reached. Given the nature of the data in MCDA applications, sensitivity analysis is important; one simple example is a comparison of the MCDA outputs when different sets of weights are used, reflecting different stakeholders’ priorities. In many cases the analysis may be reasonably robust with the overall conclusions insensitive to reasonable variations in the assumptions about the weights.

### The relationships between the design parameters, quality of care and cost

The development of PRAM exploited a wide variety of evidence from the literature and also more specific local studies and audits, as described above. Additional research was also undertaken as part of the current study to enhance the understanding of mothers’ preferences, using surveys, experience-based design and interviews with a variety of stakeholders (see Additional files 1, 2, 3, 4 and 5). Synthesising this evidence required judgement about the quality of the information. Restricting the evidence to that comparable to a Cochrane review might have enhanced the rigour of the model but many important issues would have been excluded. Instead a more flexible approach was adopted, as advocated in reviews of the implementation challenges of decision support modelling [33]. However, such an approach should not be an excuse for disregarding the nature of the evidence and each contribution to the PRAM knowledge base was considered in terms of:

- Relevance; PRAM focussed on postnatal care in the UK NHS and when examining studies from other countries the context of the health and social care system was considered.
- Rigour; the studies were categorised distinguishing major, authoritative reviews from smaller scale studies and expert opinion using published criteria [34].

Expert judgement was used to assess the evidence and summarise the effects of changes in each design parameters on the various quality domains. The process involved the use of specially recruited experienced midwives to review the literature and other evidence distilling the key relationships, for example “early discharge may have a positive impact on safe care but only if skilled community based care is provided”. These summaries were then tested in workshops with a greater range of staff, and some service user representatives, developing a consensus. Transparency is vital when adopting such an approach and stakeholders were invited to challenge the judgements and explore the effects of different assumptions [19, 35]. This was facilitated by a incorporating a simple database in the PRAM tool providing the opportunity to readily interrogate the evidence and question the implied relationships between the resource allocation decisions and the quality of care.

The evidence summaries were used to establish a set of simple sub-models capturing the essence of relationships between the design parameters and the quality domains. As in other examples of multi-criteria decision analysis [20, 21], simple linear models were used to estimate the quantitative effects of varying each design parameter *i*. The models were calibrated using judgments of the maximum beneficial value *z*_*i*_ of each parameter. The contribution of each design parameter to each quality domain was determined by the impact matrix and the full impact of all the design parameters was assumed to be a linear sum. The resultant quality scores were normalised such that if each design parameter were set at its maximum beneficial value, the score would be 100:

*i* design parameter

*n* number of design parameters

*x* value of design parameter *i* for a particular option

*z*_*i*_ maximum beneficial value of design parameter *i*

*q*(*i*,*j*) impact of design parameter i on quality domain *j*

*s*_*j*_ score for quality domain *j*

$$ {\boldsymbol{s}}_{\boldsymbol{j}}=\frac{\mathbf{100}\ {\sum}_{\boldsymbol{i}=\mathbf{1}}^{\boldsymbol{n}}\boldsymbol{q}\left(\boldsymbol{i},\boldsymbol{j}\right)\frac{{\boldsymbol{x}}_{\boldsymbol{i}}}{{\boldsymbol{z}}_{\boldsymbol{i}}}}{\sum_{\boldsymbol{i}=\mathbf{1}}^{\boldsymbol{n}}\boldsymbol{q}\left(\boldsymbol{i},\boldsymbol{j}\right)} $$

Where substantial quantitative data were available, more rigorous sub-models were incorporated based on statistical modelling, such as regression. An example was that used to estimate changing acuities: as mothers move from labour to the postnatal ward and on to discharge into the community, acuity tends to reduce such that relatively few mothers are discharged with high acuity. This transition was reflected in the acuity data collected in the NHS workforce planning study [10], providing the basis for a sub-model capturing the effects of varying the postnatal hospital-stay on the proportions of mothers and babies in the different acuity categories when discharged into the community.

Costs were estimated using standard UK NHS data [36]. A particular feature was the model of the cost of hospital-stay which distinguished the costs of the maternity services’ staff and the bed with its associated infrastructure costs. This distinction was important as the evidence suggested that the quality of care depends more on the staff-hours input than the simple length of stay [2].

### Challenging assumptions and dealing with uncertainty

As in other applications of MCDA, the process of determining the scores for each option in each quality domain or criterion involves a mixture of data: some are reasonably robust but others are obtained from expert judgment or consensus. Indeed, this capability to incorporate diverse forms of data is a distinguishing feature of MCDA. Using such data inevitably implies a significant degree of uncertainty about the precise values but the scores provide an indication of the relative strengths and weaknesses across the domains [21–23]. A fuller analysis of the component uncertainties, using Monte Carlo simulation, might quantify the overall uncertainty in the quality scores but this was not explored in the current study though it might be worthy of further work [37]. Following the practice adopted in the large majority of other MCDA applications deterministic sensitivity analysis was used instead, exploring the effects of varying key parameters in a systematic manner [37]. The sensitivity analyses were particularly important when examining assumptions and interpretations of the knowledge base, providing a mechanism for distinguishing the crucial assumptions from those that have little eventual impact on the quality scores. A prime example was the weighting to the different quality domains where sensitivity analysis helped confirm that the overall conclusion was robust given the reasonable range of variation in the allocated weights.

## Results

### An example: Reducing costs and increasing quality in a typical service

**Fig. 2:**
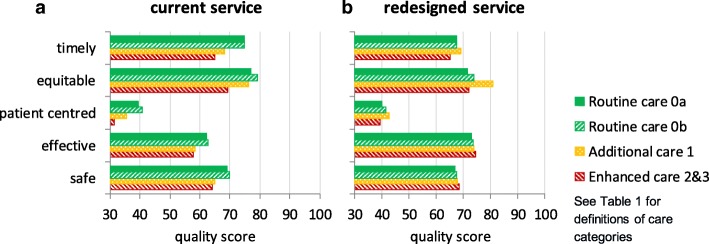
Comparing scores for each quality domain of option **a** (current provision) and **b** (a redesigned service)

**Fig. 3:**
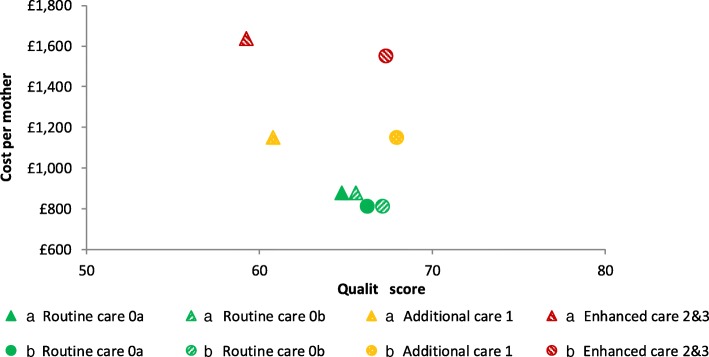
Comparing the costs and aggregate quality scores of options a (current provision) and b (a redesigned service)

The PRAM model has been used in a NHS Trust in England to support the redesign of their postnatal care, leading to significant changes and improvements in the service; other NHS organisations are also now beginning to use PRAM. A full evaluation has yet to be completed but the model appears to have improved the redesign process, with better engagement from all staff, and also enhanced outcomes, reflected in reduced readmission rates. The example described here is a simplified account of an analysis of just one redesign option; in practice many variants were explored. The current service is specified as option A in Tables 3 and 4 with the consequent quality and costs illustrated in Figs. 2 and 3. Figure 2 provides the scores for each quality domain, distinguishing the four categories of mothers and babies. Figure 3 presents the costs and the aggregate quality scores, reflecting the weights of Table 2.

**Table 3:**
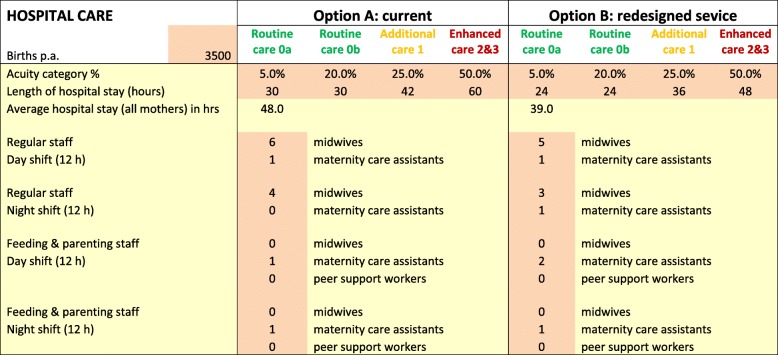
Specifying demand and staffing for hospital-based postnatal care for different care categories (see Table 1 for definitions of care categories)

**Table 4:**
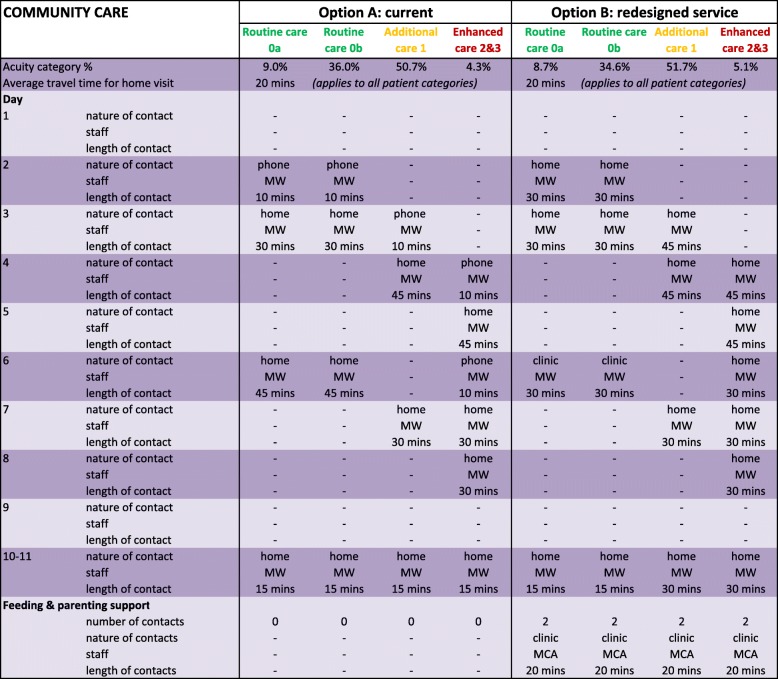
Specifying demand, pathways and staffing for community-based postnatal care (see Table 1 for definitions of care categories)

An important step in the process was the validation of this initial assessment in discussions with various staff to confirm that the scores for each quality domain of the current service, option A, were reasonable. Although the absolute quality scores employ an arbitrary scale, with 100 representing an ideal world with no resource constraints, the relative scores should reflect current experiences. The assessment of the current service, as illustrated in Figs. 2 and 3, suggested relatively low care quality for the category 1–3 mothers/ babies. This provided the focus of the efforts to redesign the service while still striving to reduce costs. One proposal was to reduce inpatient stay and enhance the community service, with a particular emphasis on the higher acuity categories. This redesigned service, designated as option B in the Tables 3 and 4, involved a package of changes to the postnatal care service including:

- reduction in hospital stay of 20% across all categories
- reduction in hospital regular staffing of 10% but an increase in dedicated feeding and parenting staff on the postnatal ward
- all mothers receive an extra home visit; category 2&3 mothers receive two extra home visits
- all mothers attend a feeding and parenting support clinic

The results of Fig. 3 suggest that the overall quality would be largely unaffected for the category 0a and 0b mothers/ babies but the categories’ 1–3 quality is increased significantly. The annual cost estimates indicate a possible saving of 7%. Figure 2 provides a more detailed consideration of the implications for each of the quality domains. In particular all categories could benefit from an increase in “effectiveness”: in this particular example the result could be classified as a potential “win-win” in the health equity impact plane [28]. Such an assessment was the first step of the decision making process with the outputs stimulating debate and the exploration of further options.

### A sensitivity analysis: Exploring the effects of changing the quality domains’ weights

**Fig. 4:**
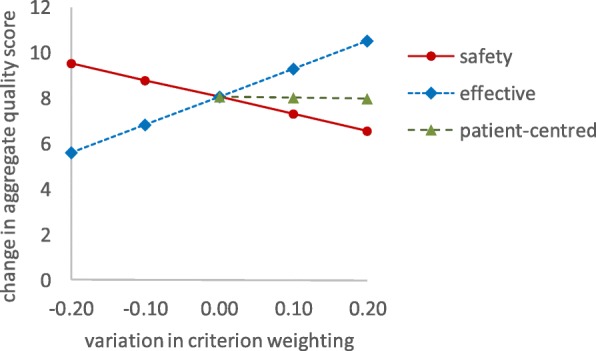
Sensitivity analysis exploring the effects of variations in the weightings of selected quality domains on the improvement in the aggregate quality scores

Given the nature of many of the data used in the analysis, it was important to determine the robustness of any recommendations with sensitivity analyses exploring the effects of varying key parameters. The weighting of the quality domains can be particularly contentious with different stakeholders having different views. The weights of Table 2 were varied in a systematic manner to explore the effects on the aggregate quality scores. The crucial issue was whether the improvements in the quality scores for the redesigned service, option B, compared to the current provision, option A, were retained. Focussing on just one key care category 2&3, the analysis suggests that the expected change in the aggregate quality score of adopting option B is an improvement of + 8.06, assuming the weights of Table 2. If the weighting associated with safety is increased by 0.1 from 0.5 to 0.6 (with the other domains’ weights being adjusted proportionally to ensure a total of 1), the improvement in the quality score is + 7.32. Figure 4 illustrates the results of exploring a range of variations in the weighting for safety and effectiveness and patient-centred by ±0.1 and ± 0.2 about the original values of Table 2. While changes in the weights inevitably affect the aggregate quality scores, the outputs are reasonably robust to such variations and the overall conclusions are not affected.

## Discussion

### Evaluating modelling interventions in health care

Ideally the value of PRAM would be determined by the actual quality outcomes for the mothers and babies, and the costs of providing the service. Measures such as readmissions may be useful but care is needed in attributing the cause of any improvement in a complex environment subject to numerous changes. Evaluations of healthcare decision support models, such as PRAM, often focus on an examination of how the model has contributed to the decision making process, typically relying on qualitative evidence [38]. As part of an ongoing study, NHS staff (midwifery staff with management responsibilities) views of their experience with PRAM were obtained and they provide some insight into their perspective of the benefits of the tool (see Additional file 7 PRAM evaluation).

### The model and its ease of use

Priority setting and option appraisal tools have been used successfully in numerous applications. However, it has been suggested [12, 24] that a combination of MCDA and PBMA might expand the scope for such tools. This approach was adopted in PRAM and it does appear to offer flexibility in its use of diverse data spanning a wide range of quality criteria. PRAM can be described as a “rapid and relevant” [35, 39, 40] mechanism for translating a summary of other research studies into practical support for redesign. Experience from its implementation suggests that the first impression of the model can be a little daunting for some staff but once the initial apprehension is overcome, the model is reasonably clear. Quotes from staff include: “Once you understand the principles, it is fascinating to see how the quality scores change in relation to different inputs”.

### The modelling process

A major benefit of modelling is that it encourages a systematic approach to the whole process of data collection, understanding the current problems and assessing redesign options [38]. The assembly of the input data required for PRAM can be a challenge, as noted by some staff there can be some “initial difficulty in marrying routinely collected clinical data with the requirements of the model.” However, staff recognised that the approach provides a discipline and a focus on the essential data: “we would not have put this data together except for PRAM”.

### Enhancing understanding

Although an obvious role of a model such as PRAM is to provide predictive assessments of effects of possible redesign and reallocation of resources, a more fundamental benefit can be in facilitating a greater shared understanding. Effective modelling requires the engagement of all staff; it also promotes engagement with staff developing a wider, shared understanding as part of a more deliberative, learning process [17, 30, 31]. PRAM emphasises the possible consequences of service changes along the whole pathway, both in hospital and the community, encouraging a fuller systemic understanding: “it makes you reflect when you’re looking at the journey as a whole”. PRAM does not always result in a radical redesign; sometimes it just provides a mechanism for communicating existing ideas in a more effective manner, as reflected in staff comments: “It helps you to articulate what you already know much better… (and) to argue your case to commissioners better”; “It has put postnatal care on the agenda”.

## Conclusions

The resource allocation model, PRAM, provided a practical decision support tool helping staff explore options and articulate their proposals for the redesign of postnatal care. The integration of cost and quality domains facilitated a variety of trade-offs, allowing staff to explore the benefits of reallocating resources, between hospital and community-based care, and between patient groups with different needs. Although the model was developed in the context of maternity care it might be adapted to examine other health care services. The flexibility, incorporating diverse evidence, and the relative ease of implementation may be valuable in many applications. However, caution is needed. In more critical services, where safety and effectiveness dominate the quality criteria, a more traditional evaluation may be needed using only the most rigorous data.

## Additional files

Additional file 1: NHS Scotland Maternity Care Survey. Copy of questionnaire capturing mothers’ experiences of maternity care in general. (PDF 1525 kb)

Additional file 2: Developing pathways. Guide for workshops and individual interviews collecting information to develop pathways. (PDF 107 kb)

Additional file 3: EBD questionnaire. Copy of questionnaire for Experience Based Design exercise relating mothers’ experiences of postnatal care to key steps in the pathways. (PDF 360 kb)

Additional file 4: Postnatal care survey. Copy of questionnaire capturing more specific data describing mothers’ experiences of postnatal care. (PDF 474 kb)

Additional file 5: Exploring assumptions. Guide for workshops and individual staff interviews checking the pathways and possible redesign actions. (PDF 575 kb)

Additional file 6: Prototype testing. Guide for workshops to gain staff feedback on the PRAM model. (PDF 328 kb)

Additional file 7: PRAM evaluation. Guide for interviews collecting staff experiences with the use of PRAM. (PDF 87 kb)

## Abbreviations

MCDA: Multicriteria decision analysis
NHS: National Health Service
PBMA: Programme budgeting and marginal analysis
PRAM: Postnatal care resource allocation model
UK: United Kingdom

## References

[1] Monitor. Closing the NHS Funding Gap: how to get better value health care for patient. Available from: https://www.gov.uk/government/publications/closing-the-nhs-funding-gap-how-to-get-better-value-healthcare-for-patients. Accessed 19 Jan 2018.

[2] Bowers J, Cheyne H. Reducing the length of postnatal hospital stay: implications for cost and quality of care. BMC Health Serv Res 2015; 16:16 doi: 10.1186/s12913-015-1214-4. Available from: https://bmchealthservres.biomedcentral.com/articles/10.1186/s12913-015-1214-4 [Accessed December 12, 2017]. 10.1186/s12913-015-1214-4 PMC4714454 26772389

[3] MacArthur C, Winter HR, Bick DE, Knowles H, Lilford RJ, Henderson C, Lancashire RJ, Braunholtz DA, Gee H (2002). Effects of redesigned community postnatal care on individual women’s health four months after birth: a cluster randomised controlled trial. Lancet.

[4] Marshall C. Modelling the shift in the balance of care in the NHS. University of Stirling PhD thesis 2013. Available from: http://hdl.handle.net/1893/20350

[5] Care Quality Commission. National findings from the 2013 survey of women’s experiences of maternity care. Available from: http://www.nhssurveys.org/Filestore/MAT13/MAT13_maternity_report_for_publication.pdf. Accessed 19 Jan 2018.

[6] Cheyne H, Skar S, Paterson A, David S, Hodgkiss F. Having a baby in Scotland 2013: Women’s experiences of maternity care, volume 1: National results Scottish Government, NHS Scotland. Available from: http://www.scotland.gov.uk/Publications/2014/01/8489 . Accessed 19 Jan 2018.

[7] Care Quality Commission. CQC’s response to the 2015 survey of women’s experiences of maternity care. Available from: https://www.cqc.org.uk/sites/default/files/20160125_maternity_survey_2015_cqc_response.pdf. Accessed 19 Jan 2018.

[8] National Institute for Health and Care Excellence. Postnatal care up to 8 weeks after birth. Available from: https://www.nice.org.uk/guidance/cg37/resources/postnatal-care-up-to-8-weeks-after-birth-975391596997. Accessed 19 Jan 2018. 32065741

[9] The Royal College of Midwives. Postnatal care planning: pressure points, the case for better postnatal care. Available from: https://www.rcm.org.uk/sites/default/files/Pressure%20Points%20-%20Postnatal%20Care%20Planning%20-%20Web%20Copy.pdf. Accessed 19 Jan 2018.

[10] NHS Education. National Midwife Workload and Workforce Planning (NMWWP) Learning Toolkit. Available from: http://www.nes.scot.nhs.uk/media/248268/nursing_midwifery_workforce_toolkit.pdf . Accessed 19 Jan 2018.

[11] Mitton C, Donaldson C (2001). Twenty-five years of programme budgeting and marginal analysis in the health sector, 1974-1999. J Health Serv Res Policy.

[12] Mitton C, Peacock S Comparative effectiveness research and priority setting. Comparative Effectiveness Research in Health Services 2016 pp 95–103. Available from: https://link.springer.com/referenceworkentry/10.1007/978-1-4899-7600-0_4. Accessed 19 Jan 2018.

[13] Tsourapas A, Frew E (2011). Evaluating ‘success’ in programme budgeting and marginal analysis: a review of the literature. J Health Serv Res Policy.

[14] Cornelissen E, Mitton C, Davidson A, Reid RC, Hole R, Visockas A, Smith N (2014). Changing priority setting practice: the role of implementation in practice change. Health Policy.

[15] Mitton CR, Donaldson C (2003). Setting priorities and allocating resources in health regions: lessons from a project evaluating program budgeting and marginal analysis (PBMA). Health Policy.

[16] Brousselle A, Lessard C (2011). Economic evaluation to inform health care decision-making: promise, pitfalls and a proposal for an alternative path. Soc Sci Med.

[17] Baltussen R, Jansen MP, Mikkelsen E, Tromp N, Bijlmakers L, van der Wilt GJ (2016). Priority setting for universal health coverage: we need evidence-informed deliberative processes, not just more evidence on cost-effectiveness. Int J Health Policy Manage.

[18] Phelps CE, Madhavan G (2017). Using multicriteria approaches to assess the value of health care. Value Health.

[19] Baltussen R, Jansen MPM, Bijlmakers L, Grutters J, Kluytmans A, Reuzel RP, Tummers M, Van der Wilt GJ (2017). Value assessment frameworks for HTA agencies: the organization of evidence –informed deliberative processes. Value Health.

[20] Baltussen R, Youngkong S, Francesco Paolucci F, Niessen L (2010). Multi-criteria decision analysis to prioritize health interventions: capitalizing on first experiences. Health Policy.

[21] Thokala P, Duenas A (2012). Multiple criteria decision analysis for health technology assessment. Value Health.

[22] Thokala P, Devlin N, Marsh K (2016). Multiple criteria decision analysis for health care decision making—an introduction:report1of the ISPOR MCDA emerging good practices task force. Value Health.

[23] Marsh K, IJzerman M, Thokala P (2016). Multiple criteria decision analysis for health care decision making—emerging good practices: report 2 of the ISPOR MCDA emerging good practices task force. Value Health.

[24] Peacock S, Mitton C, Bate A, McCoy B, Donaldson C (2009). Overcoming barriers to priority setting using interdisciplinary methods. Health Policy.

[25] Institute of Medicine (2001). Crossing the quality chasm: a new health system for the 21st century.

[26] Pickles J, Hide E, Maher L (2008). Experience based design: a practical method of working with patients to redesign services. Clin Gov Int J.

[27] Care Quality Commission. 2017 Maternity Survey: Quality and Methodology Report. Available from: http://www.cqc.org.uk/sites/default/files/20180130_mat17_qualitymethodology.pdf . Accessed 5 Jun 2018.

[28] Cookson R, Mirelman AJ, Grifin S, Asaria M, Dawkins B, Verguet S, Culyer AJ, Norheim OF (2017). Using cost-effectiveness analysis to address health equity concerns. Value Health.

[29] Teng F, Mitton C, MacKenzie J. Priority setting in the provincial health services authority: survey of key decision makers. BMC Health Serv Res. 2007; 10.1186/1472-6963-7-84. Available from: https://bmchealthservres.biomedcentral.com/articles/10.1186/1472-6963-7-84. Accessed 19 Jan 2018 10.1186/1472-6963-7-84 PMC1899487 17565691

[30] Jansen MP, Heldermann J-K, Boer B, Baltussen R (2017). Fair processes for priority setting: putting theory into practice. Int J Health Policy Manage.

[31] Angelis A, Kanavos P. Resource allocation and priority setting in health care: a multi-criteria decision analysis problem of value? Global Policy. 2016; 10.1111/1758-5899.12387. Available from: http://onlinelibrary.wiley.com/doi/10.1111/1758-5899.12387/pdf. Accessed 19 Jan 2018

[32] Wilson ECF, Peacock SJ, Ruta D (2009). Priority setting in practice: what is the best way to compare costs and benefits?. Health Econ.

[33] Cooper NJ, Sutton AJ, Ades AE, Paisley S, Jones DR (2007). Use of evidence in economic decision models: practical issues and methodological challenges. Health Econ.

[34] National Collaborating Centre for Primary Care. Postnatal Care: Routine postnatal care of women and their babies. Available from: https://www.nice.org.uk/guidance/cg37/evidence/full-guideline-485782237 . Accessed 19 Jan 2018.

[35] Philips Z, Bojke L, Sculpher M, Claxton K, Golder S (2006). Good practice guidelines for decision-analytic modelling in health technology assessment - a review and consolidation of quality assessment. PharmacoEconomics.

[36] NHS Improvement. Reference costs. Available from: https://improvement.nhs.uk/resources/reference-costs/ Accessed 15 May 2018.

[37] Broekhuizen H, Groothuis-Oudshoorn GM, van Til JA, Hummel JM, IJzerman MJ (2015). A review and classification of approaches for dealing with uncertainty in multi-criteria decision analysis for healthcare decisions. PharmacoEconomics.

[38] Weinstein MC, O'Brien B, Hornberger J, Jackson J, Johannesson M, McCabe C, Luce BR (2003). Principles of good practice for decision analytic modeling in health-care evaluation: report of the ISPOR task force on good research practices-modeling studies. Value Health.

[39] Glasgow RE, Phillips SM, Sanchez MA (2014). Implementation science approaches for integrating eHealthresearch into practice and policy. Int J Med Inform.

[40] Peek CJ, Glasgow RE, Stange KC, Klesges LM, Purcell PE, Kessler RS (2014). The 5 R’s: an emerging bold standard for conducting relevant research in a changing world. Ann Fam Med.

