# Supplementary material for: A multicriteria resource allocation model for the redesign of services following birth
Source: BMC Health Serv Res. 2018 Aug 22;18:656. doi: 10.1186/s12913-018-3430-1 (PMC6106921; doi:10.1186/s12913-018-3430-1)
Supplement: Supplementary file 1 — NHS Scotland Maternity Care Survey. Copy of questionnaire capturing mothers’ experiences of maternity care in general. (PDF 1525 kb) [file 12913_2018_3430_MOESM1_ESM.pdf]

Maternity Care Survey

Please read the enclosed letter for more information about this survey.

If you would prefer, you may also complete this survey online:

Go to www.quality- health.co.uk/sm15

Or scan the QR Code with your tablet / smartphone ➔

To do this you will need to enter the ID number which can be found on the letter which accompanies this questionnaire.

Instructions

Please answer all questions, unless the instructions ask you to skip a question.

For each question, please put a tick in the box next to the answer that most closely matches your own experience.

For example, if your answer is yes:

☑ Yes

☐ No

Don't worry if you make a mistake. Simply cross it out and tick the correct answer.

Helpline
0800 783 1775
Monday – Friday
9am – 5pm

Please return in the envelope provided or to:

Quality Health
FREEPOST RSKS-ZERR-UAGY
Unit 1, Holmewood Business Park
Chesterfield Road
Holmewood
Chesterfield
S42 5US

SECTION A. DATES AND YOUR BABY

A1. Did you give birth to a single baby, twins or more in your most recent pregnancy?

1 ☐ A single baby
2 ☐ Twins
3 ☐ Triplets, quads or more

A2. Roughly how many weeks pregnant were you when your baby was born?

1 ☐ Before I was 37 weeks pregnant
2 ☐ When I was 37 weeks pregnant or more

SECTION B. CARE WHILE YOU WERE PREGNANT (ANTENATAL CARE)

The start of your care in pregnancy

B1. Who was the **first** health professional you saw when you thought you were pregnant? **(Tick ONE only)**

1 ☐ GP / family doctor
2 ☐ Midwife
3 ☐ Other

B2. Roughly how many weeks pregnant were you when you first saw this health professional about your pregnancy care?

1 ☐ When I was 0 to 6 weeks pregnant
2 ☐ When I was 7 to 12 weeks pregnant
3 ☐ When I was 13 or more weeks pregnant
4 ☐ Don't know / Can't remember

B3. Roughly how many weeks pregnant were you when you had your 'booking' appointment (when you were given your notes, were seen by a midwife)?

1 ☐ When I was 0 to 7 weeks pregnant
2 ☐ When I was 8 or 9 weeks pregnant
3 ☐ When I was 10 or 11 weeks pregnant
4 ☐ When I was 12 weeks pregnant
5 ☐ When I was 13 or more weeks pregnant
6 ☐ Don't know / Can't remember

B4. During your pregnancy were you **offered** any of the following choices about where to have your baby? **(Tick ALL that apply)**

1 ☐ I was offered a choice of hospitals
2 ☐ I was offered a choice of giving birth in a midwife led unit or birth centre
3 ☐ I was offered a choice of giving birth in a consultant led unit
4 ☐ I was offered a choice of giving birth at home
5 ☐ I was not offered any choices
6 ☐ I had no choices due to medical reasons
7 ☐ Don't know / Can't remember

B5. Before your baby was born, did you plan to have a home birth?

1 ☐ Yes
2 ☐ No

B6. Did you get enough information from either a **midwife or doctor** to help you decide where to have your baby?

1 ☐ Yes, definitely
2 ☐ Yes, to some extent
3 ☐ No
4 ☐ No, but I did not need this information
5 ☐ Don't know / Can't remember

Antenatal check-ups

> A ‘check-up’ is any contact with a doctor or midwife to check the progress of your pregnancy. It usually includes having your blood pressure and urine checked. *Please ignore other appointments that* ***did not*** *include these things, such as a visit to the hospital for a scan or a blood test only.*

B7. During your pregnancy were you given a **choice** about **where** your antenatal check-ups would take place?

1 ☐ Yes
2 ☐ No
3 ☐ Don’t know / Can’t remember

B8. Which of the following health professionals did you see for your **antenatal check-ups**?
(Tick ALL that apply)

1 ☐ Midwife
2 ☐ GP (family doctor)
3 ☐ Hospital doctor (e.g. a consultant)
4 ☐ Other

B9. If you saw a midwife for your **antenatal check-ups**, did you see the same one every time?

1 ☐ Yes, every time
2 ☐ Most of the time
3 ☐ No
4 ☐ I only saw a midwife once
5 ☐ I did not see a midwife
6 ☐ Don’t know / Can’t remember

B10. Did you have a named midwife?

1 ☐ Yes, and I saw them all / most of the time
2 ☐ Yes, and I saw them some of the time
3 ☐ Yes, but I did not see them
4 ☐ No
5 ☐ Don’t know / Can’t remember

B11. During your antenatal check-ups, were you given enough time to ask questions or discuss your pregnancy?

1 ☐ Yes, always
2 ☐ Yes, sometimes
3 ☐ No
4 ☐ Don’t know / Can't remember

B12. During your antenatal check-ups, were you listened to?

1 ☐ Yes, always
2 ☐ Yes, sometimes
3 ☐ No
4 ☐ Don’t know / Can’t remember

B13. During your antenatal check-ups, were your personal circumstances taken into account?

1 ☐ Yes, definitely
2 ☐ Yes, to some extent
3 ☐ No
4 ☐ Don’t know / Can’t remember

B14. **During your pregnancy,** did you have a discussion with a Health Visitor?

1 ☐ Yes, and it was useful
2 ☐ Yes, but it was not useful
3 ☐ No, but I would have found it useful
4 ☐ No, but I did not want to
5 ☐ Don’t know / Can’t remember

During your pregnancy

B15. During your pregnancy, did you have a telephone number for a midwife or midwifery team that you could contact?

1 ☐ Yes
2 ☐ No
3 ☐ Don't know / Can't remember

B16. During your pregnancy, if you contacted a midwife or the midwifery team, were you given the help you needed?

1 ☐ Yes, always
2 ☐ Yes, sometimes
3 ☐ No
4 ☐ No, as I was not able to contact a midwife or the midwifery team
5 ☐ I did not contact a midwife or the midwifery team

B17. Thinking about your **antenatal care**, were you spoken to in a way you could understand?

1 ☐ Yes, always
2 ☐ Yes, sometimes
3 ☐ No
4 ☐ Don't know / Can't remember

B18. Thinking about your **antenatal care**, were you involved enough in decisions about your care?

1 ☐ Yes, always
2 ☐ Yes, sometimes
3 ☐ No
4 ☐ I did not want / need to be involved
5 ☐ Don't know / Can't remember

B19. Overall, how would you rate your **antenatal care**?

1 ☐ Excellent
2 ☐ Good
3 ☐ Fair
4 ☐ Poor
5 ☐ Very poor

If there is anything else you would like to tell us about your antenatal care, please do so here.

SECTION C. YOUR LABOUR AND THE BIRTH OF YOUR BABY

Note: If you had a planned caesarean please go to Question C6

C1. At the very start of your labour, did you feel that you were given appropriate advice and support when you contacted a midwife or the hospital?

1 ☐ Yes
2 ☐ No
3 ☐ I did not contact a midwife or the hospital

C2. During your labour, were you able to move around and choose the position that made you most comfortable?

1 ☐ Yes, most of the time
2 ☐ Yes, sometimes
3 ☐ No, not at all
4 ☐ No, but it was not possible due to medical reasons / No, but I didn't want to.

C3. During your pregnancy, were you given enough information about the pain relief you could use when giving birth?

1 ☐ Yes, definitely

2 ☐ Yes, to some extent

3 ☐ No, but I would have found it useful

4 ☐ No, but I did not want / need information

5 ☐ Dont know / Can't remember

C4. Did you feel that you had enough help to enable you to cope with your pain during labour?

1 ☐ Yes, always

2 ☐ Yes, most of the time

3 ☐ No

4 ☐ I did not want / need help

The birth of your baby

C5. Where was your baby born?

1 ☐ In hospital

2 ☐ At home

3 ☐ Other (please state)

C6. Thinking about the birth of your baby, what **type of delivery** did you have? (*If you had twins or more than two babies this time, please fill in this question about the baby who was born first*)

1 ☐ A normal vaginal delivery ➔ **Go to C7**

2 ☐ An assisted vaginal delivery (e.g. with forceps or ventouse suction cup) ➔ **Go to C7**

3 ☐ A planned caesarean delivery ➔ **Go to C9**

4 ☐ An emergency caesarean delivery ➔ **Go to C9**

C7. Where did you give birth? **(Tick ONE only)**

1 ☐ On a bed

2 ☐ On a mat on the floor

3 ☐ In a water or birthing pool

4 ☐ Other

C8. What position were you in **when your baby was born? (Tick ONE only)**

1 ☐ Sitting / sitting supported by pillows

2 ☐ On my side

3 ☐ Standing, squatting or kneeling

4 ☐ Lying flat / lying supported by pillows

5 ☐ Lying with legs in stirrups

6 ☐ Other

C9. Did you have skin to skin contact (*baby naked, directly on your chest or tummy*) with your baby shortly after the birth?

1 ☐ Yes

2 ☐ Yes, but I did not want this

3 ☐ No

4 ☐ No, but this was not possible for medical reasons

5 ☐ No, I did not want skin to skin contact with my baby

The staff caring for you

C10. Did the staff caring for you introduce themselves?

1 ☐ Yes, all of the staff introduced themselves

2 ☐ Some of the staff introduced themselves

3 ☐ Very few or none of the staff introduced themselves

4 ☐ Don’t know / Can’t remember

C11. If your partner or someone else close to you was involved in your care during labour and birth, were they able to be involved as much as they wanted?

1 ☐ Yes
2 ☐ No
3 ☐ They did not want to be involved
4 ☐ I did not want them to be involved
5 ☐ I did not have a partner or a companion with me

C12. Were you (and / or your partner or a companion) left alone by midwives or doctors at a time when it worried you?
(Tick ALL that apply)

1 ☐ Yes, during early labour
2 ☐ Yes, during the later stages of labour
3 ☐ Yes, during the birth
4 ☐ Yes, shortly after the birth
5 ☐ No, not at all

C13. If you raised a concern **during labour and birth,** did you feel that it was taken seriously?

1 ☐ Yes
2 ☐ No
3 ☐ I did not raise any concerns.

C14. When you called / asked for assistance **during labour and birth,** did you receive it within a reasonable time?

1 ☐ Yes, always
2 ☐ Yes, sometimes
3 ☐ No
4 ☐ I didn't need / want assistance
5 ☐ Don’t know / Can’t remember

C15. Thinking about your **care during labour and birth**, were you spoken to in a way you could understand?

1 ☐ Yes, always
2 ☐ Yes, sometimes
3 ☐ No
4 ☐ Don’t know / Can’t remember

C16. Thinking about your **care during labour and birth**, were you involved enough in decisions about your care?

1 ☐ Yes, always
2 ☐ Yes, sometimes
3 ☐ No
4 ☐ I did not want / need to be involved
5 ☐ Don’t know / Can’t remember

C17. Thinking about your **care during labour and birth**, were you treated with respect and dignity?

1 ☐ Yes, always
2 ☐ Yes, sometimes
3 ☐ No
4 ☐ Don’t know / Can’t remember

C18. Did you have confidence and trust in the staff caring for you during your labour and birth?

1 ☐ Yes, definitely
2 ☐ Yes, to some extent
3 ☐ No
4 ☐ Don’t know / Can’t remember

C19. Overall, how would you rate the care you received during your labour and birth?

1 ☐ Excellent
2 ☐ Good
3 ☐ Fair
4 ☐ Poor
5 ☐ Very poor

If there is anything else you would like to tell us about **your labour and birth,** please do so here.

SECTION D. CARE IN HOSPITAL AFTER THE BIRTH (POSTNATAL CARE)

Note: If you had a home birth and did not go to hospital, please go to question E1

D1. How long did you stay in hospital after your baby was born?

1 ☐ Up to 12 hours
2 ☐ More than 12 hours but less than 24 hours
3 ☐ 1 to 2 days
4 ☐ 3 to 4 days
5 ☐ 5 or more days

D2. Looking back, do you feel that the length of your stay in hospital after the birth was...

1 ☐ About right
2 ☐ Too long
3 ☐ Too short
4 ☐ Not sure / Don’t know

D3. Thinking about the care you received in hospital after the birth of your baby, were you given the information or explanations you needed?

1 ☐ Yes, always
2 ☐ Yes, sometimes
3 ☐ No
4 ☐ Don’t know / Can’t remember

D4. Thinking about the care you received in hospital after the birth of your baby, were you treated with kindness and understanding?

1 ☐ Yes, always
2 ☐ Yes, sometimes
3 ☐ No
4 ☐ Don’t know / Can’t remember

D5. Thinking about your stay in hospital, if your partner or someone else close to you was involved in your care, were they able to stay with you as much as you wanted?
(Tick ALL that apply)

1 ☐ Yes
2 ☐ No, as they were restricted to visiting hours
3 ☐ No, as there was no accommodation for them in hospital
4 ☐ No, they were not able to stay for another reason
5 ☐ I did not have a partner or companion with me

D6. Thinking about your stay in hospital, how clean was the hospital room or ward you were in?

1 ☐ Very clean
2 ☐ Fairly clean
3 ☐ Not very clean
4 ☐ Not at all clean
5 ☐ Don’t know / Can’t remember

D7. Thinking about your stay in hospital, how clean were the toilets and bathrooms you used?

1 ☐ Very clean
2 ☐ Fairly clean
3 ☐ Not very clean
4 ☐ Not at all clean
5 ☐ Don’t know / Can’t remember
6 ☐ I did not use the toilet / bathroom

D8. Overall, how would you rate the care you received in hospital after the birth?

1 ☐ Excellent
2 ☐ Good
3 ☐ Fair
4 ☐ Poor
5 ☐ Very poor

If there is anything else you would like to tell us about about **your care in hospital after the birth,** please do so here.

SECTION E. FEEDING YOUR BABY

E1. During your pregnancy did **midwives or the midwifery team** provide relevant information about feeding your baby?

1 ☐ Yes, definitely
2 ☐ Yes, to some extent
3 ☐ No
4 ☐ I did not want / need this information
5 ☐ Don't know / Can't remember

E2. In the first few days after the birth how was your baby fed? **(Tick ONE only)**

1 ☐ Breast milk (or expressed breast milk) only **➔ Go to E4**
2 ☐ Both breast and formula (bottle) milk **➔ Go to E4**
3 ☐ Formula (bottle) milk only **➔ Go to E3**
4 ☐ Other **➔ Go to E3**
5 ☐ Not sure **➔ Go to E3**

E3. Did you ever try to breastfeed your baby (even if it was only once)?

1 ☐ Yes
2 ☐ No

E4. Were your decisions about how you wanted to feed your baby respected by staff?

1 ☐ Yes, always
2 ☐ Yes, sometimes
3 ☐ No
4 ☐ Don't know / Can't remember

E5. Did you feel that midwives and other health professionals gave you **consistent advice** about **feeding your baby**?

1 ☐ Yes, always
2 ☐ Yes, sometimes
3 ☐ No
4 ☐ I did not want or need any advice
5 ☐ I did not receive any advice
6 ☐ Don't know / Can't remember

E6. Did you feel that midwives and other health professionals gave you active **support and encouragement** about **feeding your baby**?

1 ☐ Yes, always
2 ☐ Yes, sometimes
3 ☐ No
4 ☐ I did not want / need this
5 ☐ Don't know / Can't remember

SECTION F. CARE AT HOME AFTER THE BIRTH

F1. When you were at home after the birth of your baby, did you have a telephone number for a midwife or midwifery team that you could contact?

1 ☐ Yes
2 ☐ No
3 ☐ Don't know / Can't remember

F2. If you contacted a midwife or midwifery team were you given the help you needed?

1 ☐ Yes, always
2 ☐ Yes, sometimes
3 ☐ No
4 ☐ No as I was not able to contact a midwife
5 ☐ I did not contact a midwife

F3. Since your baby's birth have you been visited at home by a midwife?

1 ☐ Yes
2 ☐ Yes, but I had to contact them to arrange this
3 ☐ No, this was not offered
4 ☐ No, I was visiting or staying near my baby in a neonatal unit (NNU, NICU, SCBU)
5 ☐ No, for another reason

F4. Since your baby's birth have you seen a midwife at a clinic?

1 ☐ Yes ➔ **Go to F5**
2 ☐ Yes, but I had to contact them to arrange this ➔ **Go to F5**
3 ☐ No, this was not offered ➔ **Go to F6**
4 ☐ No , I was visiting or staying near my baby in a neonatal unit (NNU, NICU, SCBU) ➔ **Go to F6**
5 ☐ No, for another reason ➔ **Go to F6**

F5. Was it convenient for you to see a midwife at a clinic?

1 ☐ Yes, completely
2 ☐ Yes, to some extent
3 ☐ Not at all
4 ☐ Don't know / Not applicable

F6. Were you given a choice about **where** you saw a midwife?

1 ☐ Yes
2 ☐ No
3 ☐ Don't know / Can't remember

F7. If you saw a midwife for your care at home or in a clinic, after birth, did you see the same one every time?

1 ☐ Yes, every time
2 ☐ Yes, most of the time
3 ☐ No
4 ☐ I only saw a midwife **once**
5 ☐ Don't know / Can't remember

F8. Did you see your named midwife at home or in a clinic after birth?

1 ☐ I did not have a named midwife
2 ☐ Yes, I saw them all / most of the time
3 ☐ Yes, I saw them some of the time
4 ☐ No
5 ☐ Don't know / Can't remember

Thinking about all the times you were visited at home or seen in a clinic by a midwife after the birth…

F9. How many times in total did you see a midwife after you went home?

1 ☐ 1 - 2
2 ☐ 3 - 4
3 ☐ 5 - 6
4 ☐ 7 times or more
5 ☐ Don't know / Can't remember

F10. Would you have liked to have seen a midwife...

1 ☐ More often?
2 ☐ Less often?
3 ☐ I saw a midwife as much as I wanted

F11. Did you feel that the **midwife** or **midwives** that you saw always listened to you?

1 ☐ Yes, always
2 ☐ Yes, sometimes
3 ☐ No
4 ☐ Don't know / Can't remember

F12. Did the midwife or midwives that you saw take your personal circumstances into account when giving you advice?

1 ☐ Yes, always
2 ☐ Yes, sometimes
3 ☐ No
4 ☐ This was not necessary
5 ☐ Don't know / Can't remember

F13. Did you have confidence and trust in the midwives and midwifery team you saw after going home?

1 ☐ Yes, always
2 ☐ Yes, sometimes
3 ☐ No
4 ☐ Don't know / Can't remember

F14. Did you see the same midwife for both your antenatal and postnatal care?

1 ☐ Yes, always
2 ☐ Yes, most of the time
3 ☐ No, but I would have liked to
4 ☐ No, but I did not mind
5 ☐ Don't know / Can't remember

F15. In the six weeks after the birth of your baby did you receive help and advice from health professionals about your **baby's health and progress**?

1 ☐ Yes, definitely
2 ☐ Yes, to some extent
3 ☐ No
4 ☐ I did not want or need any advice
5 ☐ Don't know / Can't remember

F16. In the six weeks after the birth of your baby did you receive help and advice from a midwife or health visitor about **feeding your baby**?

1 ☐ Yes, definitely
2 ☐ Yes, to some extent
3 ☐ No
4 ☐ I did not want or need any advice
5 ☐ Don't know / Can't remember

F17. Did a midwife tell you that you would need to arrange a postnatal check-up of your own health? (Around 4 - 8 weeks after the birth)

1 ☐ Yes
2 ☐ No
3 ☐ Don't know / Can't remember

F18. Were you given enough information about your own physical recovery after the birth?

1 ☐ Yes, definitely
2 ☐ Yes, to some extent
3 ☐ No
4 ☐ No, but I did not need this information
5 ☐ Don't know / Can't remember

F19. Were you given enough information about any emotional changes you might experience after the birth?

1 ☐ Yes, definitely
2 ☐ Yes, to some extent
3 ☐ No
4 ☐ No, but I did not need this information
5 ☐ Don't know / Can't remember

F20. Were you told who you could contact if you needed advice about emotional changes you might experience after the birth?

1 ☐ Yes
2 ☐ No
3 ☐ Don't know / Can't remember

F21. Were you given information or offered advice from a health professional about contraception?

1 ☐ Yes
2 ☐ No
3 ☐ Don't know / Can't remember

F22. Overall, how would you rate the care you received at home after the birth?

1 ☐ Excellent
2 ☐ Good
3 ☐ Fair
4 ☐ Poor
5 ☐ Very poor

If there is anything else you would like to tell us about **your postnatal care** please do so here.

SECTION G. ABOUT YOU

Your answers will help us to describe the women taking part in the survey and to find out if different groups of women have different experiences of their maternity care. If you would prefer not to answer a particular question then you can miss it out.

G1. What was your **age** on your last birthday?

G2. Have you had a previous pregnancy?

1 ☐ Yes ➔ **Go to G3**
2 ☐ No ➔ **Go to G4**

G3. How many babies have you given birth to before this pregnancy?

1 ☐ None
2 ☐ 1-2
3 ☐ 3 or more

G4. How would you rate your health in general?

1 ☐ Very good
2 ☐ Good
3 ☐ Fair
4 ☐ Bad
5 ☐ Very bad

G5. Do you have a physical or mental health condition or illness lasting or expected to last 12 months or more?

1 ☐ Yes ➔ **Go to G6**
2 ☐ No ➔ **Go to G7**

G6. Does your condition or illness reduce your ability to carry-out day-to-day activities?

1 ☐ Yes, a lot
2 ☐ Yes, a little
3 ☐ Not at all

G7. What religion, religious denomination or body do you belong to?

1 ☐ None
2 ☐ Church of Scotland
3 ☐ Roman Catholic
4 ☐ Other Christian
5 ☐ Muslim
6 ☐ Buddhist
7 ☐ Sikh
8 ☐ Jewish
9 ☐ Hindu
10 ☐ Pagan
11 ☐ Another religion (non-Christian)

G8. Which of the following best describes how you think of yourself?

1 ☐ Heterosexual / straight
2 ☐ Gay / lesbian
3 ☐ Bisexual
4 ☐ Other

G9. What is your **ethnic group**?

Tick **ONE** box which **best describes** your ethnic group

1 ☐ White
2 ☐ Mixed or multiple ethnic groups
3 ☐ Asian, Asian Scottish or Asian British
4 ☐ African
5 ☐ Caribbean or Black
6 ☐ Other ethnic group

G10. In which NHS Board did all or most of your antenatal care take place? **(Tick ONE only)**

1 ☐ Ayrshire and Arran
2 ☐ Borders
3 ☐ Dumfries and Galloway
4 ☐ Fife
5 ☐ Forth Valley
6 ☐ Greater Glasgow and Clyde
7 ☐ Grampian
8 ☐ Highland
9 ☐ Lanarkshire
10 ☐ Lothian
11 ☐ Orkney
12 ☐ Shetland
13 ☐ Tayside
14 ☐ Western Isles
15 ☐ Other (e.g. outwith Scotland, abroad)
16 ☐ Don't know

NHS Statisticians hold information about your stay in hospital. We would like your permission to add your survey results to this information. Your information will be used only for research and will not identify you individually.

If you give your permission to add your survey results to this information it will not be shared with the people who looked after you and will in no way affect your current or future treatment or care.

G11. Do you give your permission for NHS Statisticians to add your survey results to information held about your hospital stay?

1 ☐ Yes
2 ☐ No

G12. We may be conducting further research in order that we can learn more about the experiences of patients. Would you like to be considered as a participant in this future research?

1 ☐ Yes, I would like to take part in any future research
2 ☐ No, I would not like to take part in any future research

If you are interested in participating, we would like to contact you by email. Please provide your email address if you are happy for us to do this.

THANK YOU VERY MUCH FOR YOUR HELP

Please post this questionnaire back in the FREEPOST envelope provided.

No stamp is needed.
