# Supplementary material for: A multicriteria resource allocation model for the redesign of services following birth
Source: BMC Health Serv Res. 2018 Aug 22;18:656. doi: 10.1186/s12913-018-3430-1 (PMC6106921; doi:10.1186/s12913-018-3430-1)
Supplement: Supplementary file 2 — Developing pathways. Guide for workshops and individual interviews collecting information to develop pathways. (PDF 107 kb) [file 12913_2018_3430_MOESM2_ESM.pdf]

Developing postnatal care pathways

1. Postnatal care in the community may involve activities other than face-to-face visits, e.g. does the mother have the opportunity to phone for advice?
2. Need to distinguish staff and the roles of people other than midwives, e.g. care assistants, GP's, district nurses: all may contribute to postnatal care.
3. How does postnatal care interact with other services?
4. How is a mother and baby's postnatal care organised, and by whom:
    - 4.1. If risk factors/ needs have been identified during antenatal or intrapartum care how (when and whose responsibility) do these affect the design of the postnatal care?
    - 4.2. If there are major changes in need during postnatal care how is the postnatal care reorganised?
    - 4.3. If there are minor variations in mother/ baby's needs are these usually provided within the flexibility of routine postnatal care, e.g. the number and frequency of discretionary midwife visits (day 4 and days 6-9 visits). How do individual midwives make these decisions: are they provided with much guidance?
    - 4.4. How do midwives (and other staff involved in postnatal care) plan their day/ week? How is workload allocated? This planning may be particularly important in community postnatal care where there may be a trade-off between efficiency and quality (e.g. continuity) of care. Safety would not be jeopardised but there may be marginal aspects of quality that have to be sacrificed?
5. The information flows associated with the organisational decisions of 4 should be mapped. The form of the information should be distinguished, e.g. computer based record, paper record and word-of-mouth. This may be especially important where different staff (see 2) and services (see 3) are involved.
6. Information flows and decision points should be included as switchable features on the Visio pathways: they may be a critical detail but could obscure the big picture if they are always visible.
