# Supplementary material for: A multicriteria resource allocation model for the redesign of services following birth
Source: BMC Health Serv Res. 2018 Aug 22;18:656. doi: 10.1186/s12913-018-3430-1 (PMC6106921; doi:10.1186/s12913-018-3430-1)
Supplement: Supplementary file 3 — EBD questionnaire. Copy of questionnaire for Experience Based Design exercise relating mothers’ experiences of postnatal care to key steps in the pathways. (PDF 360 kb) [file 12913_2018_3430_MOESM3_ESM.pdf]

Participant number

The questions below ask about how you felt in the few hours and days after your baby was born. For each of the questions please circle the number that most applied to you. For each question 1 is the worst and 5 the best you might have felt.

In the first few hours after my baby was born

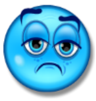

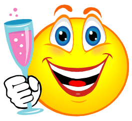

I felt very unwell	1	2	3	4	5	I felt very well
I felt very tired	1	2	3	4	5	I didn't feel tired at all
I was in a lot of pain	1	2	3	4	5	I felt no pain at all
I was not supported by staff at all	1	2	3	4	5	I was very well supported by staff
I felt very under confident about caring for my baby	1	2	3	4	5	I felt very confident about caring for my baby
I had no help or advice about caring for my baby	1	2	3	4	5	I had all the help and advice I needed to help me care for my baby

By the end of my stay in hospital

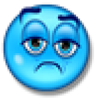

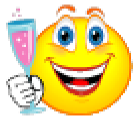

I felt very unwell	1	2	3	4	5	I felt very well
I felt very tired	1	2	3	4	5	I didn't feel tired at all
I was in a lot of pain	1	2	3	4	5	I felt no pain at all
I was not supported by staff at all	1	2	3	4	5	I was very well supported by staff
I felt very under confident about caring for my baby	1	2	3	4	5	I felt very confident about caring for my baby
I had no help or advice about caring for my baby	1	2	3	4	5	I had all the help and advice I needed to help me care for my baby

After the first night home with my baby

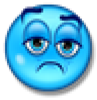

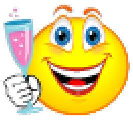

I felt very unwell	1	2	3	4	5	I felt very well
I felt very tired	1	2	3	4	5	I didn’t feel tired at all
I was in a lot of pain	1	2	3	4	5	I felt no pain at all
I was not supported by staff at all	1	2	3	4	5	I was very well supported by staff
I felt very under confident about caring for my baby	1	2	3	4	5	I felt very confident about caring for my baby
I had no help or advice about caring for my baby	1	2	3	4	5	I had all the help and advice I needed to help me care for my baby

After the first visit by the midwife

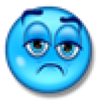

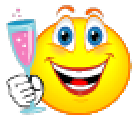

I felt very unwell	1	2	3	4	5	I felt very well
I felt very tired	1	2	3	4	5	I didn't feel tired at all
I was in a lot of pain	1	2	3	4	5	I felt no pain at all
I was not supported by staff at all	1	2	3	4	5	I was very well supported by staff
I felt very under confident about caring for my baby	1	2	3	4	5	I felt very confident about caring for my baby
I had no help or advice about caring for my baby	1	2	3	4	5	I had all the help and advice I needed to help me care for my baby

At the time the midwife stopped seeing me

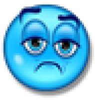

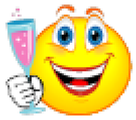

I felt very unwell	1	2	3	4	5	I felt very well
I felt very tired	1	2	3	4	5	I didn't feel tired at all
I was in a lot of pain	1	2	3	4	5	I felt no pain at all
I was not supported by staff at all	1	2	3	4	5	I was very well supported by staff
I felt very under confident about caring for my baby	1	2	3	4	5	I felt very confident about caring for my baby
I had no help or advice about caring for my baby	1	2	3	4	5	I had all the help and advice I needed to help me care for my baby
