# Supplementary material for: A multicriteria resource allocation model for the redesign of services following birth
Source: BMC Health Serv Res. 2018 Aug 22;18:656. doi: 10.1186/s12913-018-3430-1 (PMC6106921; doi:10.1186/s12913-018-3430-1)
Supplement: Supplementary file 4 — Postnatal care survey. Copy of questionnaire capturing more specific data describing mothers’ experiences of postnatal care. (PDF 474 kb) [file 12913_2018_3430_MOESM4_ESM.pdf]

CHIEF SCIENTIST OFFICE
Nursing, Midwifery and Allied Health Professions Research Unit

PRAM
Postnatal Care Resource Allocation Model

PARTICIPANT NUMBER

POSTNATAL MATERNITY CARE SURVEY

What is the survey about?

The Postnatal Maternity Care Survey is part of a larger study that is looking at how postnatal care is delivered in the United Kingdom. It is examining how postnatal care is currently being delivered and looking at ways to improve the service for women and their families. As part of the study we want to understand your experience of postnatal care. Your views are very important in helping us find out how good the services are and how they can be improved.

Completing the questionnaire

Please only think about the postnatal care you received following your **most recent** birth when answering these questions.

For most questions, please tick clearly inside one box ☑ using a black or blue pen. For some questions you may be asked to tick more than one box.

Not all sections will apply to you. Sometimes you will find the box you have ticked has an instruction to go to another question. By following the instructions carefully you will miss out questions that do not apply to you.

Taking part in this survey is voluntary. Your answers will be treated in confidence and will not be shared with the health professionals who looked after you.

If the survey raises issues or questions of concern, you may wish to contact your family doctor (GP) or Health Visitor.

If you have any questions please contact Dr Miranda Page or Dr Helen Cheyne at

NMAHP Research Unit
Iris Murdoch Building
University of Stirling
Stirling
FK9 4LA
Tel: 01786 466462 Email m.e.page@stir.ac.uk or h.l.cheyne@stir.ac.uk

PART ONE OF THE POSTNATAL MATERNITY CARE SURVEY

SECTION A. DATES AND YOUR BABY

A1. When was your baby born? (*If you had twins or more than two babies this time, please fill in this question about the baby who was born first*)

Day | Month | Year

A2. What time was your baby born? (*If you had twins or more than two babies this time, please fill in this question about the baby who was born first*)

1 ☐ Early morning (12:01am-6:00am)
2 ☐ Morning (6:01am-12:00 noon)
3 ☐ Afternoon (12:01pm-6:00pm)
4 ☐ Evening / Night (6:01pm-12:00 midnight)

A3. Roughly how many weeks pregnant were you when your baby was born?

1 ☐ Before I was 37 full weeks pregnant
2 ☐ When I was 37 weeks pregnant or more

A4. How much did your **baby weigh at birth**? (*If you had twins or more than two babies this time, please fill in this question about the baby who was born first*)

1 ☐ Less than 2500g / 2.5kg (Less than 5 pounds 8 ounces)
2 ☐ 2500g / 2.5kg or more (5 pounds 8 ounces or more)
3 ☐ Don’t know / Can’t remember

The birth of your baby

C1. Where was your baby born?

1 ☐ In hospital labour ward/labour suite
2 ☐ In a birth centre/maternity unit, separate from hospital
3 ☐ In a birth centre/maternity unit, within a maternity hospital
4 ☐ Other

C2. Thinking about the birth of your baby, what **kind of delivery** did you have? (*If you had twins or more than two babies this time, please fill in this question about the baby who was born first*)

1 ☐ A normal vaginal delivery
2 ☐ An assisted vaginal delivery (e.g. with forceps or ventouse suction cup)
3 ☐ A planned caesarean delivery ➔ **Go to E1**
4 ☐ An emergency caesarean delivery ➔ **Go to E1**

C3. While your baby was being born were you given an **episiotomy (cut)**?

1 ☐ Yes
2 ☐ No

C4. While your baby was being born did you have a **tear**?

1 ☐ Yes ➔ **Go to C5**
2 ☐ No ➔ **Go to E1**

C5. Was this a **serious tear** which involved your back passage (third or fourth degree tear)?

1 ☐ Yes
2 ☐ No

SECTION E CARE IN HOSPITAL AFTER THE BIRTH (POSTNATAL CARE)

E1. How long did you stay in hospital after your baby was born?

1 ☐ Up to 12 hours
2 ☐ More than 12 hours but less than 24 hours
3 ☐ 1 to 2 days
4 ☐ 3 to 4 days
5 ☐ 5 or more days

E2. Looking back, do you feel that the length of your stay in hospital after the birth was...

1 ☐ Too long
2 ☐ Too short
3 ☐ About right
4 ☐ Not sure / Don’t know

E3. Was there a member of staff available to help you during your stay?

1 ☐ Yes, always
2 ☐ Yes, sometimes
3 ☐ No
4 ☐ Don’t know / Can’t remember

E4. Thinking about the care you received in hospital after the birth of your baby, were you given the information or explanations you needed?

1 ☐ Yes, always
2 ☐ Yes, sometimes
3 ☐ No
4 ☐ Don't know / Can't remember

E5. Thinking about the care you received in hospital after the birth of your baby, were you spoken to in a way you could understand?

1 ☐ Yes, always
2 ☐ Yes, sometimes
3 ☐ No
4 ☐ Don't know / Can't remember

E6. Thinking about the care you received in hospital after the birth of your baby, were you treated with respect and dignity?

1 ☐ Yes, always
2 ☐ Yes, sometimes
3 ☐ No
4 ☐ Don't know / Can't remember

E7. Did your baby have a newborn examination or 'baby check' before you were discharged home?

1 ☐ Yes ➔ **Go to E8**
2 ☐ No ➔ **Go to E9**
3 ☐ Don't know / Can't remember ➔ **Go to E9**

E8. Who carried out this examination or 'baby check'? (Tick ONE only)

1 ☐ Midwife
2 ☐ Doctor (e.g. a paediatrician)
3 ☐ Other
4 ☐ Don't know / Can't remember

E9. Overall, how would you rate the care received during your stay in hospital after the birth of your baby?

1 ☐ Excellent
2 ☐ Very good
3 ☐ Good
4 ☐ Fair
5 ☐ Poor

SECTION F. FEEDING YOUR BABY

F1. During your pregnancy did your midwife discuss infant feeding with you?

1 ☐ Yes, definitely
2 ☐ Yes, to some extent
3 ☐ No
4 ☐ Don't know / Can't remember

F2. In the first few days after the birth how was your baby fed? (Tick ONE only)

1 ☐ Breast milk (or expressed breast milk) only ➔ **Go to F4**
2 ☐ Both breast and formula (bottle) milk ➔ **Go to F4**
3 ☐ Formula (bottle) milk only ➔ **Go to F3**
4 ☐ Not sure ➔ **Go to F3**

F3. Did you ever put your baby to the breast (even if it was only once)?

1 ☐ Yes
2 ☐ No

Thinking about feeding your baby (breast or bottle) in the <u>first few days</u> after the birth…

F4. Did you feel that midwives and other carers gave you **consistent advice**?

1 ☐ Yes, always
2 ☐ Yes, generally
3 ☐ No
4 ☐ Don't know
5 ☐ I didn't want or need this

F5. Did you feel that midwives and other carers gave you **practical help when you needed it**?

1 ☐ Yes, always
2 ☐ Yes, generally
3 ☐ No
4 ☐ Don't know
5 ☐ I didn't want or need this

F6. Did you feel that midwives and other carers gave you active **support and encouragement**?

1 ☐ Yes, always
2 ☐ Yes, generally
3 ☐ No
4 ☐ Don't know
5 ☐ I didn't want or need this

SECTION H. CARE AT HOME AFTER THE BIRTH

H1. When you were at home after the birth of your baby did you have the name and telephone number of a midwife or health visitor you could contact if you were worried?

1 ☐ Yes
2 ☐ No
3 ☐ Don't know / Can't remember

H2. If you contacted a midwife or health visitor, were you given the help you needed?

1 ☐ Yes, always ➔ **Go to H3**
2 ☐ Yes, sometimes ➔ **Go to H3**
3 ☐ No ➔ **Go to H3**
4 ☐ I did not contact a midwife or health visitor ➔ **Go to H4**

H3. When you contacted a midwife, did you get a response as soon as you needed it?

1 ☐ Yes, always
2 ☐ Yes, sometimes
3 ☐ No
4 ☐ Don't know / Can't remember

H4. Since your baby's birth have you been visited at home by a midwife?

1 ☐ Yes ➔ **Go to H5**
2 ☐ No, I visited the midwife or saw a midwife in a clinic ➔ **Go to H5**
3 ☐ No, I was not offered a visit ➔ **Go to H8**
4 ☐ No, I was visiting or staying near my baby in a neonatal unit (NNU, NICU, SCBU) ➔ **Go to H8**
5 ☐ No, for another reason ➔ **Go to H8**

H5. How many times in total did you see a midwife after you went home?

1 ☐ 1-2
2 ☐ 3-4
3 ☐ 5-6
4 ☐ 7 times or more
5 ☐ Don't know / Can't remember

H6. Would you have liked to have seen a midwife...

1 ☐ More often?
2 ☐ Less often?
3 ☐ I saw a midwife as much as I wanted

H7. Did you have confidence and trust in the midwives you saw after going home?

1 ☐ Always
2 ☐ Sometimes
3 ☐ Rarely
4 ☐ Never

H8. Since your baby's birth have you been visited at home by a maternity support worker?

1 ☐ Yes ➔ **Go to H9**
2 ☐ No ➔ **Go to H10**

H9. How many times in total did you see a maternity support worker after you went home?

1 ☐ 1-2
2 ☐ 3-4
3 ☐ 5-6
4 ☐ 7 times or more
5 ☐ Don't know / Can't remember

H10. Where would you have liked to have seen or contacted a midwife or maternity support worker for postnatal care of you and your baby? (Tick ALL that apply)

1 ☐ My home
2 ☐ Regular clinic based at my GP's surgery
3 ☐ Open drop-in baby clinic
4 ☐ By phone

H11. Were you given enough information about your own recovery after the birth?

1 ☐ Yes, definitely
2 ☐ Yes, to some extent
3 ☐ No
4 ☐ No, but I did not need this information
5 ☐ Don't know / Can't remember

H12. Were you given enough information about any emotional changes you might experience after the birth?

1 ☐ Yes, definitely
2 ☐ Yes, to some extent
3 ☐ No
4 ☐ No, but I did not need this information
5 ☐ Don't know / Can't remember

H13. Were you given information or offered advice from a health professional about contraception?

1 ☐ Yes
2 ☐ No
3 ☐ Don't know / Can't remember

H14. Overall, how would you rate the care received **<u>at home</u> after the birth?**

1 ☐ Excellent
2 ☐ Very good
3 ☐ Good
4 ☐ Fair
5 ☐ Poor

PART TWO - HEALTH AND WELLBEING AFTER THE BIRTH (POSTNATAL)

Part two of the survey asks questions about your health and wellbeing in the days and weeks following the birth of your baby.

1. Were you able to get enough rest during your stay in hospital?

1 ☐ Yes
2 ☐ No
3 ☐ Sometimes

2. Below is a list of symptoms some women experience after having a baby. Please tick any you might have experienced.

1 ☐ Fever/shivering/high temperature
2 ☐ Excessive vaginal loss
3 ☐ Offensive vaginal loss
4 ☐ Severe or persistent headache
5 ☐ Perineal pain (area between your back passage and vagina)
6 ☐ Haemorrhoids (piles)
7 ☐ Constipation
8 ☐ Difficulty passing urine
9 ☐ Caesarean section wound problems
10 ☐ Cracked or painful nipples
11 ☐ Engorged breasts
12 ☐ Mastitis (inflammation / infection of breast)
13 ☐ Other breastfeeding problems

3. If you didn't experience any of these symptoms or you can't remember please go to question 8

4. Did you have difficulty talking to a midwife about these health symptoms?

1 ☐ Yes ➔**Go to 5**
2 ☐ No ➔ **Go to 8**

5. How many of these health symptoms were you able to talk to your midwife about?
(Tick ONE only)

1 ☐ None
2 ☐ A few
3 ☐ Some of them
4 ☐ Most of them
5 ☐ All of them

6. Did you talk to anyone else about any of these health symptoms? (please tick all that apply)

1 ☐ No
2 ☐ Maternity care assistant
3 ☐ Support worker
3 ☐ GP
4 ☐ Health visitor
5 ☐ Friend
6 ☐ Relative
7 ☐ Other (please say who)
....

7. Why did you find it difficult to speak to a member of staff? (please tick all that apply)

1 ☐ Staff were too busy
2 ☐ I was too busy
3 ☐ I was too embarrassed
4 ☐ Problem was not bad enough to take up professional's time
5 ☐ I felt the professionals could not help
6 ☐ Other (please say who)
....

8. After having a baby some women need emotional support. Did you feel that you needed support emotionally?

1 ☐ No ➔ **Go to 11**
2 ☐ Yes ➔ ➔ **Go 9**

9. Did you have any difficulties talking to a health professional about the emotional support you needed?

1 ☐ No ➔ **Go to 11**
2 ☐ Yes ➔ **Go to 10**

10. Why do you think this was? (tick all that apply)

1 ☐ Staff were too busy

2 ☐ I was too busy

3 ☐ I was too embarrassed

4 ☐ Problem was not bad enough to take up professional's time

5 ☐ I felt the professionals could not help

6 ☐ Other (please say what)…………………………………..

11. Thinking about when you left hospital. Did you feel you had enough information to care for yourself and your baby?

1 ☐ Yes ➔ **Go to 13**

2 ☐ No ➔ **Go to 12**

3 ☐ Don't know ➔ **Go to 13**

12. What would you have liked (more) information on? (Please tick **all** those that apply)

1 ☐ Care of baby (e.g., bathing, changing nappy

2 ☐ Use of car seats to take baby home

3 ☐ Health problems for babies

4 ☐ Health problems in women after giving birth

5 ☐ Family planning

6 ☐ Breastfeeding

7 ☐ Formula (bottle) feeding

8 ☐ The neonatal blood spot

9 ☐ More details on follow up in the community

10 ☐ Who to contact if a problem arose

11 ☐ How to contact a health professional

12 ☐ Other (please state what)

....

13. **During your stay in the hospital**, counting up every day and night how many midwives do you think looked after you?

1 ☐ 1

2 ☐ 2

3 ☐ 3

4 ☐ more than 3

5 ☐ Don't know

SECTION 2. HEALTH AT HOME AFTER THE BIRTH

14. After you went home with your baby, did **<u>you</u>** need to be re-admitted to hospital?

1 ☐ Yes ➔ **Go to 15**

2 ☐ No ➔ **Go to 17**

15. Why were you re-admitted?

……………………………………………………………

16. How long were you in hospital for?

………………………………………………..

17. After you went home with your baby, did **your baby** need to be re-admitted to hospital?

1 ☐ Yes ➔ **Go to 18**

2 ☐ No ➔ **Go to 20**

18. Was your baby admitted because of any of the following; (tick **all** that apply)

1 ☐ Jaundice

2 ☐ Weight loss

3 ☐ Feeding problems

4 ☐ Other

5 ☐ Don't know

19. How long was your baby in hospital for?

………………………………………………..

20. When you were at home after the birth of your baby did the GP visit you at home?

1 ☐ Yes ➔ **Go to 21**

2 ☐ No ➔ **Go to 22**

3 ☐ Don't know / Can't remember ➔ **Go to 22**

21. How many times did the GP visit?

1 ☐ 1

2 ☐ 2

3 ☐ more than 2

4 ☐ Don't know / Can't remember

22. When you were at home after the birth of your baby did the health visitor visit you at home?

1 ☐ Yes ➔ **Go to 23**

2 ☐ No ➔ **Go to 24**

3 ☐ Don't know / Can't remember➔ **Go to 24**

23. How many times did the health visitor visit?

1 ☐ 1

2 ☐ 2

3 ☐ more than 2

4 ☐ Don't know / Can't remember

SECTION 3. WELLBEING AT HOME AFTER THE BIRTH

These questions are asking how you feel now

24. How would you say you are feeling physically?

1 ☐ Very well
2 ☐ Reasonably well
3 ☐ Neither well or unwell
4 ☐ Not very well
5 ☐ Not well at all

25. How would you say you are feeling emotionally?

1 ☐ Happy
2 ☐ Neither up nor down
3 ☐ Slightly tearful
4 ☐ Tearful
5 ☐ Very tearful

26. How would you rate your level of tiredness?

1 ☐ Not tired
2 ☐ slightly tired
3 ☐ Tired
4 ☐ Very tired

For this section please tick the answer which comes closest to how you have felt IN THE PAST WEEK, not just today.
Please answer each question

27. I have been able to laugh and see the funny side of things:

1 ☐ As much as I always could
2 ☐ Not quite so much now
3 ☐ Definitely not so much now
4 ☐ Not at all

28. I have looked forward with enjoyment to things:

1 ☐ As much as I ever did
2 ☐ Rather less than I used to
3 ☐ Definitely less than I used to
4 ☐ Hardly at all

29. I have blamed myself unnecessarily when things went wrong:

1 ☐ Yes, most of the time
2 ☐ Yes, some of the time
3 ☐ Not very often
4 ☐ No, never

30. I have been anxious or worried for no good reason:

1 ☐ No, not at all
2 ☐ Hardly ever
3 ☐ Yes, sometimes
4 ☐ Yes, very often

31. I have felt scared or panicky for no very good reason:

1 ☐ Yes, quite a lot
2 ☐ Yes, sometimes
3 ☐ No, not much
4 ☐ No, not at all

32. Things have been getting on top of me:

1 ☐ Yes, most of the time I haven't been able to cope at all
2 ☐ Yes, sometimes I haven't been coping as well as usual
3 ☐ No, most of the time I have coped quite well
4 ☐ No, I have been coping as well as ever remember

33. I have been so unhappy that I have had difficulty sleeping:

1 ☐ Yes, most of the time
2 ☐ Yes, sometimes
3 ☐ Not very often
4 ☐ No, not at all

34. I have felt sad or miserable:

1 ☐ Yes, most of the time
2 ☐ Yes, quite often
3 ☐ Not very often
4 ☐ No, not at all

35. I have been so unhappy that I have been crying:

1 ☐ Yes, most of the time
2 ☐ Yes, quite often
3 ☐ Only occasionally
4 ☐ No, never

36. The thought of harming myself has occurred to me:

1 ☐ Yes, quite often
2 ☐ Sometimes
3 ☐ Hardly ever
4 ☐ Never

Reference:
Cox JL et al., 1987

SECTION J. YOU AND YOUR HOUSEHOLD

This information will help us find out if different groups of people have different experiences of treatment during the postnatal period. Nobody at the hospital you attended will be able to see your answers. **If you would prefer not to answer a particular question then you should skip it and go to the next question.**

J1. Have you had a previous pregnancy?

1 ☐ Yes ➔ **Go to J2**
2 ☐ No ➔ **Go to J3**

J2. How many babies have you given birth to before this pregnancy?

1 ☐ None
2 ☐ 1-2
3 ☐ 3 or more

J3. How would you rate your **health** in general? *Please tick **ONE** box only*

1 ☐ Good
2 ☐ Fair
3 ☐ Poor

J4. Do you have any of the following? *Please tick **ALL** that apply*

1 ☐ Deafness or severe hearing impairment
2 ☐ Blindness or severe vision impairment
3 ☐ A Physical disability
4 ☐ A learning disability
5 ☐ A Mental health condition
6 ☐ Chronic pain lasting at least 3 months
7 ☐ Another long-term condition
8 ☐ None of the above

J5. Are your **day-to-day activities limited** because of a health problem or disability which has lasted, or is expected to last, **at least 12 months**? (Include problems related to old age) *Please tick **ONE** box*

1 ☐ Yes, limited a lot
2 ☐ Yes, limited a little
3 ☐ No

J6. What was your age last birthday?

____

J7. What best describes your **work status**? *Please tick **ONE** box only*

1 ☐ Work full time
2 ☐ Work part time
3 ☐ In full time education or training
4 ☐ Unemployed/looking for work
5 ☐ Don't work due to illness or disability
6 ☐ Other

J8. Do you look after, or give any regular help or support to family members, friends, neighbours or others because of either long-term physical/mental ill-health/disability or problems related to old age? *Exclude any caring that is done as part of any paid employment.*

1 ☐ No
2 ☐ Yes, up to 19 hours a week
3 ☐ Yes, 20-34 hours a week
4 ☐ Yes, 35 or more hours a week

J9. Which of the following options best describes how you think of yourself? *Please tick **ONE** box only*

1 ☐ Heterosexual / Straight
2 ☐ Homosexual/ Gay or Lesbian
3 ☐ Bisexual
4 ☐ Other

J10. Do you need an **interpreter** or other **help to communicate**? *Please tick **ONE** box only*

1 ☐ No
2 ☐ Yes – what type of help do you need?

____

J11. What **religion**, religious denomination or body do you belong to?

*Please tick **ONE** box only*

1 ☐ None
2 ☐ Church of Scotland
3 ☐ Roman Catholic
4 ☐ Church of England
5 ☐ Other Christian (please write in)

____

6 ☐ Muslim
7 ☐ Buddhist
8 ☐ Sikh
9 ☐ Jewish
10 ☐ Hindu
11 ☐ Pagan
12 ☐ Another religion (non-Christian, please write in:

____

J12. What is your **ethnic group**?

*Tick **ONE** box which **best describes** your ethnic group*

1 ☐ White
2 ☐ Mixed or multiple ethnic groups
3 ☐ Asian, Asian Scottish or Asian British
4 ☐ African, Caribbean or Black
5 ☐ Other ethnic group

If you would be willing to consider taking part in further research conducted by us about your views or experiences please put your name and address in the box below. This information will not be shared with any other organisation or third party.

Your name:

Your address:

Postcode:

SECTION K. OTHER COMMENTS

If there is anything else you would like to tell us about your maternity care, please do so here.

THANK YOU VERY MUCH FOR YOUR HELP

Please check that you answered all the questions that apply to you. Please post this questionnaire back in the PRE-PAID envelope provided. No stamp is needed
