# Supplementary material for: A multicriteria resource allocation model for the redesign of services following birth
Source: BMC Health Serv Res. 2018 Aug 22;18:656. doi: 10.1186/s12913-018-3430-1 (PMC6106921; doi:10.1186/s12913-018-3430-1)
Supplement: Supplementary file 5 — Exploring assumptions. Guide for workshops and individual staff interviews checking the pathways and possible redesign actions. (PDF 575 kb) [file 12913_2018_3430_MOESM5_ESM.pdf]

PRAM Workshop – discussion group outline questions

The following are meant as guidance – hopefully we will be able to have a fairly unstructured discussion that will arise naturally from the STOP, START and CONTINUE exercise. Gill's questions might come up naturally within the discussion, but they are pasted below as a reminder.

1. We have identified some key elements of postnatal care that work really well. From your own service, what do you think works particularly well that could be implemented in other settings?
2. We have identified some areas that need to be improved. What might be the best way of solving these issues?

Need to work through some of the key problem areas that participants highlighted in the S, S & C exercise and try to get them to identify solutions or alternative modes of service delivery. Some general areas to keep in mind:

- In hospital issues: care of women with particular needs e.g. C-Sections
- Infant feeding/Breastfeeding support – in hospital and at home
- Wound/infection care – in hospital and at home
- Information handover – in hospital to community midwife to health visitor
- Working across sectors e.g. midwifery, health visiting, social work
- Mental health care in both hospital and community
- Infants with special needs?
- Young mothers/addiction problems etc involving cross agency involvement?

(Mother/ baby care flow) To what extent are the KCND pathways adhered to in practice?

(Mother/ baby care flow) What constraints are there on the discharge procedure due to staff shift times? E.g. is there often a delay in arranging prescriptions?

(Community) Can some home visits be replaced by clinics? What is the best way of teaching mothers about baby care, feeding, parent craft (hospital ward, at home or leaflets)?

(Community) What are people's feelings regarding the quality of postnatal care provided to mothers living in geographically isolated areas? More generally, is there any way to improve communication to mothers regarding the timing of home visits?

(Community) Are there any difficulties associated with the hand over between the community midwife and the public health visitor (e.g., regarding paperwork)?

(Community) To what extent are maternity care assistants able to carry out the tasks of a community midwife?

(Information) The flow of information appears fairly complicated. Is it possible to cut down on time spent on paperwork through the use of IT? Do community midwives have enough information on their first home visit?
