# Supplementary material for: A multicriteria resource allocation model for the redesign of services following birth
Source: BMC Health Serv Res. 2018 Aug 22;18:656. doi: 10.1186/s12913-018-3430-1 (PMC6106921; doi:10.1186/s12913-018-3430-1)
Supplement: Supplementary file 6 — Prototype testing. Guide for workshops to gain staff feedback on the PRAM model. (PDF 328 kb) [file 12913_2018_3430_MOESM6_ESM.pdf]

PRAM prototype testing

The workshop is intended to serve two main purposes:

a) provide feedback on the design of PRAM and identify possible improvements;
b) complete an illustrative initial analysis which can be developed in future sessions (assuming that no major issues are identified in (a).

1. Objectives of the session

1.1. Demonstrate the use of PRAM and gain feedback to revise the model.
1.2. Get the group to use the model with our help.
1.3. Compare current postnatal care design with a possible alternative system of care.
1.4. Evaluate the model's role and potential value in design and decision making; obtain feedback to refine the model.
1.5. Agree follow-up sessions which will explore more realistic alternatives.

2. Introduction

2.1. Check participants' awareness of PRAM: its objectives and principles.
2.2. Brief introduction to the project and model (adapted to reflect the audience prior knowledge).
2.3. Present the key screens of the model.

3. Working with the model

3.1. Get staff to try out the model: start by entering unit statistics and working through the data entry sheet. Fill out the pathways for each category of mother. The proposed category descriptions will be distributed ahead of the meeting to check that these are relevant to this Board.
3.2. First enter the current model of care (as option A) and check the cost and quality outcomes.
3.3. Try out different models (enter as option B) and compare the cost and quality outcomes.

4. Evaluation and outcomes

4.1. How did people find using the model generally? Explore: understanding of the model, its usability, e.g. could anything be added to better prepare people for using the model?
4.2. Did the model help to make decisions and set priorities? Explore: were there any other benefits to using the model, e.g. highlighted gaps in capacity or service provision, etc.
4.3. What are the model's limitations?
4.4. Should anything be added to the model?
4.5. Are the categories of women useful?
4.6. Who is needed to be brought into using the model or consulted on the process of change? Explore: best ways of engaging a range of stakeholders; using the model to demonstrate the rationale/ benefits of change, etc.
