# Supplementary material for: A multicriteria resource allocation model for the redesign of services following birth
Source: BMC Health Serv Res. 2018 Aug 22;18:656. doi: 10.1186/s12913-018-3430-1 (PMC6106921; doi:10.1186/s12913-018-3430-1)
Supplement: Supplementary file 7 — PRAM evaluation. Guide for interviews collecting staff experiences with the use of PRAM. (PDF 87 kb) [file 12913_2018_3430_MOESM7_ESM.pdf]

Assessing the effectiveness of the model

Requirement	Criterion	Comment
Ease of use	Practical/ accessible	
	Simplicity	
	Effort: benefits	
Effective process	Systematic	
	Transparent	
	Credible	
	Inclusive engaging diverse staff/ stakeholders	
Enhancing understanding	Shares & synthesises data	
	Evidence based	
	Incorporates value judgements	
	Highlights key, relevant criteria	
	Sufficient predictive accuracy	
	Considers different population groups	
